# DHX9-mediated pathway contributes to the malignant phenotype of myelodysplastic syndromes

**DOI:** 10.1016/j.isci.2023.106962

**Published:** 2023-05-25

**Authors:** Nanfang Huang, Yang Song, Wenhui Shi, Juan Guo, Zheng Zhang, Qi He, Lingyun Wu, Xiao Li, Feng Xu

**Affiliations:** 1 Department of Hematology, Shanghai Sixth People’s Hospital Affiliated to Shanghai Jiao Tong University School of Medicine, Shanghai 200233, China

**Keywords:** Pathophysiology, Medical Microbiology, Cancer

## Abstract

DHX9 is a member of the DEAH (Asp-Glu-Ala-His) helicase family and regulates DNA replication and RNA processing. DHX9 dysfunction promotes tumorigenesis in several solid cancers. However, the role of DHX9 in MDS is still unknown. Here, we analyzed the expression of *DHX9* and its clinical significance in 120 MDS patients and 42 non-MDS controls. Lentivirus-mediated *DHX9*-knockdown experiments were performed to investigate its biological function. We also performed cell functional assays, gene microarray, and pharmacological intervention to investigate the mechanistic involvement of DHX9. We found that overexpression of *DHX9* is frequent in MDS and associated with poor survival and high risk of acute myeloid leukemia (AML) transformation. DHX9 is essential for the maintenance of malignant proliferation of leukemia cells, and DHX9 suppression increases cell apoptosis and causes hypersensitivity to chemotherapeutic agents. Besides, knockdown of *DHX9* inactivates the PI3K-AKT and ATR-Chk1 signaling, promotes R-loop accumulation, and R-loop-mediated DNA damage.

## Highlights

• DHX9 is highly expressed in MDS and is associated with poor outcome
• DHX9-depleted inhibits PI3K-AKT and ATR-Chk1 pathways
• DHX9-depleted promotes R-loop formation and DNA damage

## Graphical abstract

**Figure:**
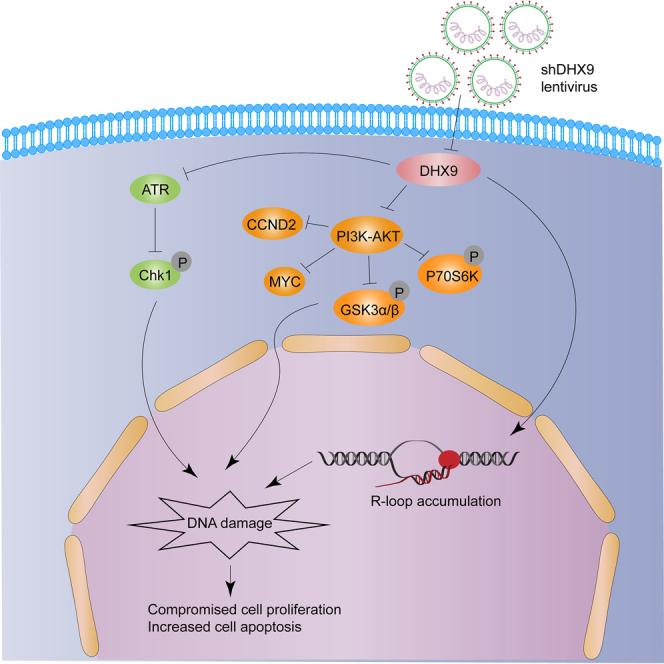

## Introduction

Myelodysplastic syndrome (MDS) is a highly heterogeneous myeloid neoplastic disease, characterized by ineffective hematopoiesis and a high risk of acute myeloid leukemia (AML) transformation.^1^ Recently, massively parallel sequencing in MDS has revealed some high-frequency mutations including RNA splicing-related genes such as SF3B1, U2AF1, and SRSF2.^2^^,^^3^^,^^4^ Functional studies of these genes have furthered our understanding of MDS pathogenesis and the development of more effective targeted treatment strategies.^5^^,^^6^^,^^7^ In a previous study, we performed whole-exome sequencing of three MDS patients and found a *DHX9* mutation in one patient.^8^ Further validated sequencing showed that most of *DHX9* mutations were defined as single nucleotide polymorphism according to the comparison of sequencing in oral mucosa epithelial cells (unpublished data). However, we unexpectedly found that *DHX9* mRNA is over-expressed in MDS patients, indicating the close association between *DHX9* and MDS development. Functionally, DHX9 as an NTP-dependent DExH/D-box helicase can unwind both RNA and DNA strands.^9^ It regulates gene expression and RNA selective splicing by interaction with transcription factors or complexes.^10^ MDS are diseases with abnormal RNA splicing, which prompts us to investigate the role of DHX9 in MDS. Additionally, two recent studies have shown that dysfunction of DDX41 and DDX3X (DHX9 members) are frequently found in leukemia and natural killer/T-cell (NKT) lymphomas, respectively, which further suggests that abnormalities in the RNA helicase family contributes to the development of hematological malignancies.^11^^,^^12^ To date, no DHX9*-*related studies have been reported in MDS. Here, we investigated the *DHX9* expression and its relationship with the clinical characteristics. We also conducted a series of functional experiments in myeloid tumor cell lines to investigate the effects of DHX9 on the biological characteristics of MDS.

## Results

### Patients’ characteristics

The MDS group was composed of 120 MDS patients with a median age of 55 years (11–84 years), which included 11 RCUD (refractory cytopenia with unilineage dysplasia), 4 RARS (refractory anemia with ringed sideroblasts), 53 RCMD (refractory cytopenia with multilineage dysplasia), 2 MDS-U (myelodysplastic syndrome, unclassified), 26 RAEB-1 (refractory anemia with excess blasts-1), and 23 RAEB-2 (refractory anemia with excess blasts-2) (Table 1). RCUD, RARS, RCMD, and MDS-U are defined as low-grade MDS (percentage of blasts <5%), while RAEB-1 and RAEB-2 are defined as high-grade MDS (percentage of blasts ≥5%). The control group contained a total of 42 cases with non-clonal cytopenias, including 16 patients with megaloblastic anemia, 8 patients with idiopathic thrombocytopenic purpura, 7 patients with iron deficiency anemia, and 11 patients with anemia of chronic diseases. Their median age was 52 years (24–91 years). No difference was observed in age or gender between MDS and control groups. Clinical information of MDS patients was shown in Table S1.

**Table 1:** Clinical characteristics of MDS patients

Variable	Total (n=120)
Sex ratio (male: female)	62:58
Median age, years (range)	55(11-84)
WHO classification	
Low-grade MDS	
RCUD	11 (9.2%)
RARS	4 (3.3%)
RCMD	53 (44.2%)
MDS-U	2 (1.7%)
MDS with 5q-	1 (0.8%)
High-grade MDS	
RAEB-1	25 (20.8%)
RAEB-2	24 (20.0%)
IPSS-R category	
Very low	2 (1.7%)
Low	24 (20.0%)
Intermediate	49 (40.8%)
High	26 (21.7%)
Very high	19 (15.8%)
Abnormal chromosomes	
5q-/-5	6 (5.0%)
20q-/-20	4 (3.3%)
Trisomy 8	8 (6.7%)
7q-/-7	5 (4.2%)
Complex karyotypes	8 (6.7%)
others	12 (10.0%)
Normal	77 (64.2%)
Median survival time (months)	undefined
AML transformation (%)	22 (18.3%)

### Increased *DHX9* expression is common in MDS and associated with poor clinical outcome

**Figure 1:**
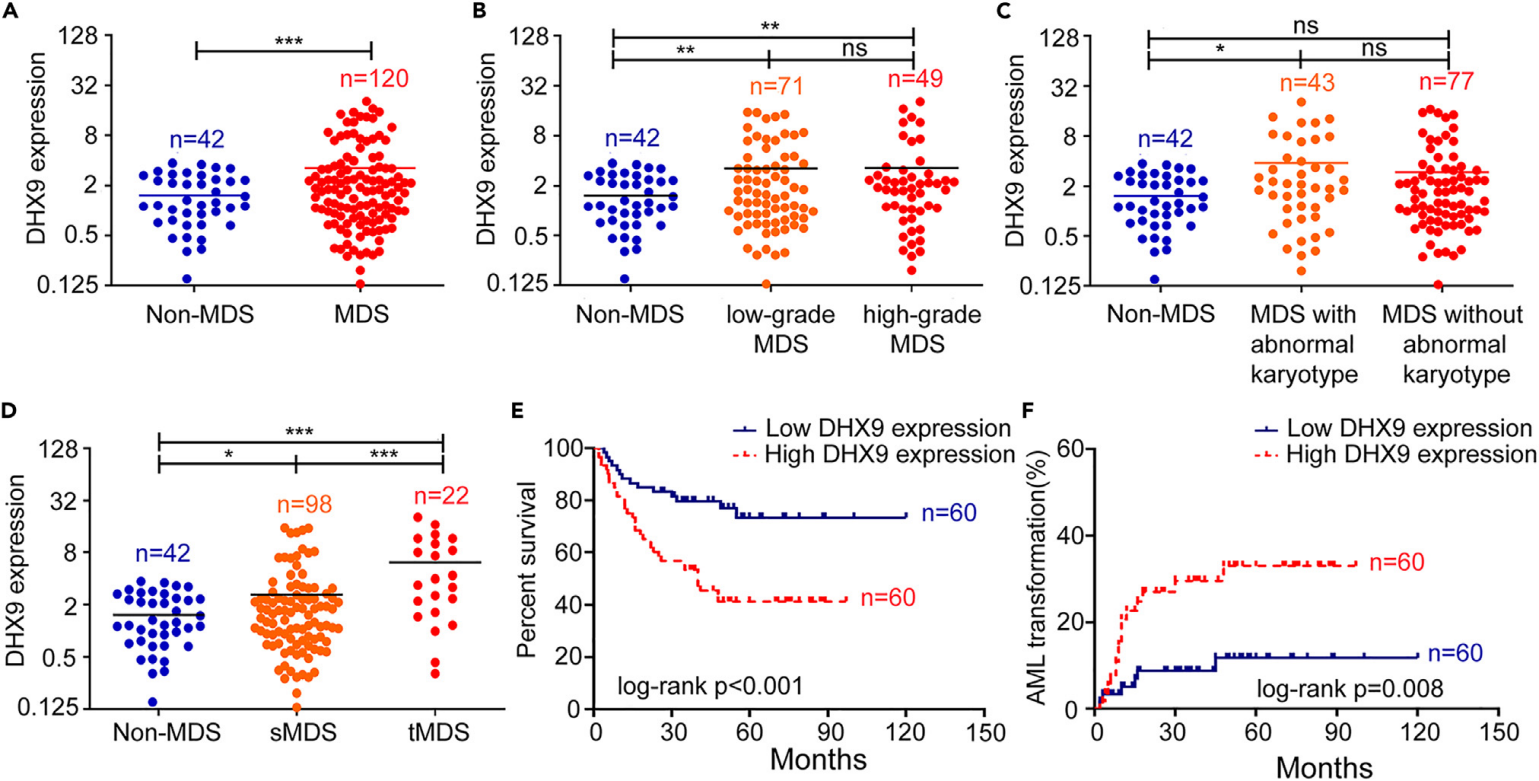
Expression analysis of *DHX9* and its effect on the survival and AML transformation in MDS patients (A) Compared with the non-MDS group, the *DHX9* expression level is higher in MDS patients. (B) Both low-grade and high-grade patients exhibits higher *DHX9* expression than non-MDS controls. (C) The *DHX9* expression is higher in patients with abnormal karyotype than non-MDS controls. (D) The patients with advanced MDS show higher *DHX9* expression than those with stable MDS. (E and F) The patients with low *DHX9* expression display a longer OS and lower AML transformation compared with those with high *DHX9* expression. The corresponding statistical analysis relative to the control group is annotated with an asterisk. ∗: p < 0.05; ∗∗: p < 0.01; ∗∗∗: p < 0.001 (two-tailed, student's t-test or Log-rank test). Data are represented as mean ± SEM.

*DHX9* mRNA levels were determined by qRT-PCR in 120 MDS patients (at the time of diagnosis) and 42 control individuals. *DHX9* expression was significantly higher in the MDS patients compared with the control individuals (p *< 0.001*) (Figure 1A). According to 2008 WHO classification, both low-grade and high-grade MDS group showed higher expression of *DHX9* than the control individuals (p = 0.001; p = 0.009) (Figure 1B). The patients with abnormal karyotype showed higher expression of *DHX9* than non-MDS controls (p = 0.031) (Figure 1C). Grouped according to disease progression, the patients with tMDS (finally transformed into RAEB or AML) showed higher *DHX9* expression than those with sMDS (stable MDS, not transformed into AML) (p *<* 0.001) (Figure 1D). To investigate the influence of *DHX9* expression on overall survival and AML transformation , we divided patients into two groups: low *DHX9* expression (≤ median value) and high *DHX9* expression (> median value). Survival analysis showed that the group with high *DHX9* expression had shorter overall survival and more risk of AML transformation compared to the group with low *DHX9* expression (p < 0.001, Figure 1E; p = 0.008, Figure 1F).

### DHX9 is indispensable for the maintenance of malignant proliferation of leukemia cells

**Figure 2:**
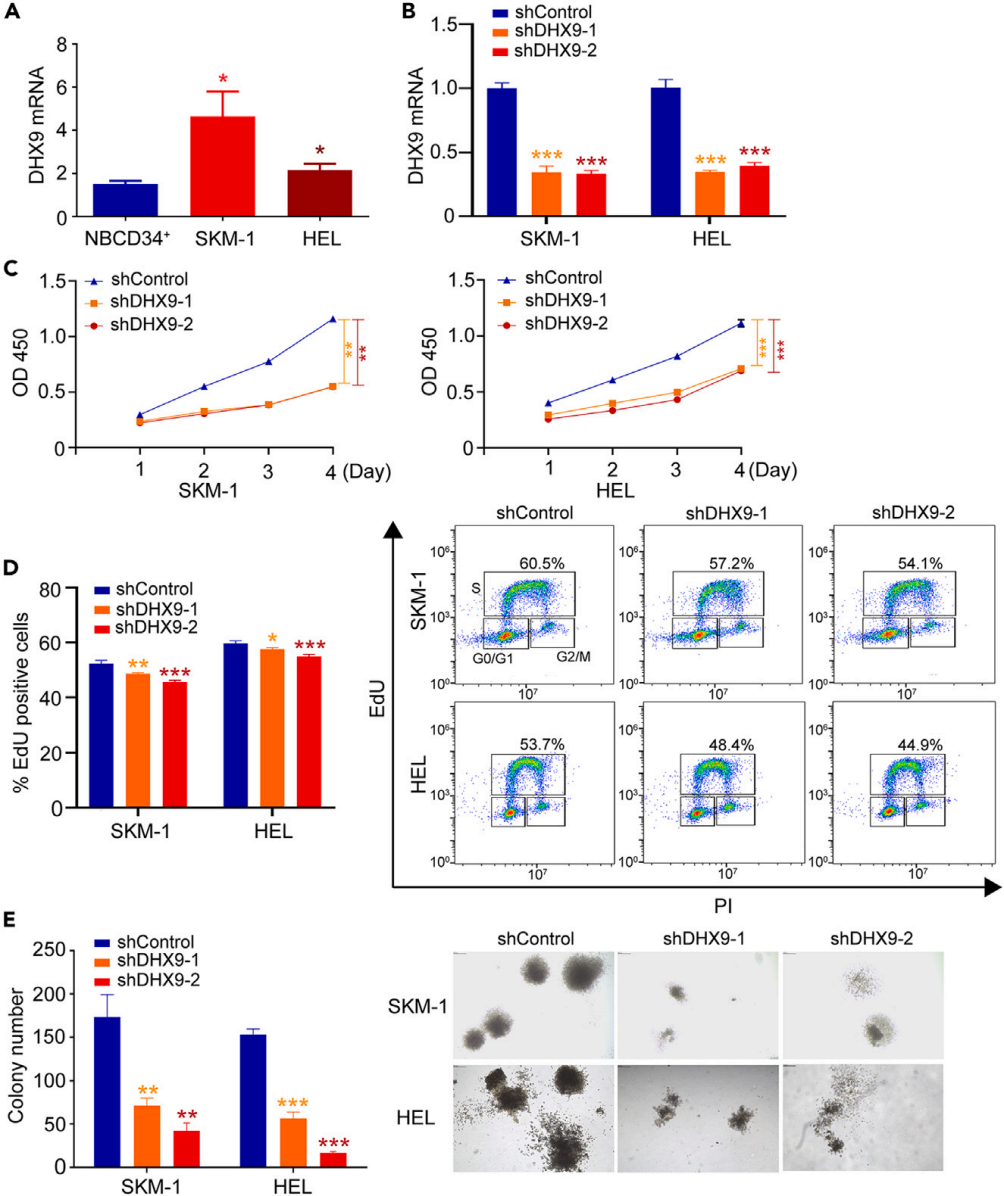
The effect of *DHX9* knockdown on the growth of myeloid tumor cell lines (A) High *DHX9* mRNA level is observed in SKM-1 and HEL cells. (B) The *DHX9* mRNA expression is detected three days after *DHX9* knockdown. (C) Growth curves show that knockdown of *DHX9* leads to reduced cell growth in the leukemia cell lines. (D) Knockdown of *DHX9* results in reduced cell percentage with EdU positive staining. (E) Reduced colony formation capacity is observed in the *DHX9*-knockdown cells compared with the control cells (40X). ∗: p < 0.05; ∗∗: p < 0.01; ∗∗∗: p < 0.001 (two-tailed, student's t-test or one-way ANOVA test). Data are represented as mean ± SEM.

qRT-PCR analysis showed that the *DHX9* mRNA level was higher in SKM-1 and HEL cells than that in the normal CD34 positive cells (Figure 2A). Two effective *DHX9*-shRNAs and control shRNA were transfected into leukemia cells, respectively. Over 60% decreases in *DHX9* expression were observed (Figure 2B). Cell counting assay indicated that *DHX9*-depleted cells exhibited reduced cell growth compared to the control cells (Figure 2C). The EdU uptake assay indicated that the cells with *DHX9* knockdown had a reduced uptake capacity of EdU compared with the control cells after treatment with 10 μM EdU for 2 h (Figure 2D). Methylcellulose assay showed that reduced colony formation was observed in the *DHX9*-knockdown cells compared with the control cells (Figure 2E). Together, these assays revealed that knockdown of *DHX9* may impair the cell proliferation capacity in the malignant hematopoietic cells.

### *DHX9* suppression increases cell apoptosis and causes hypersensitivity to chemotherapeutic agents

**Figure 3:**
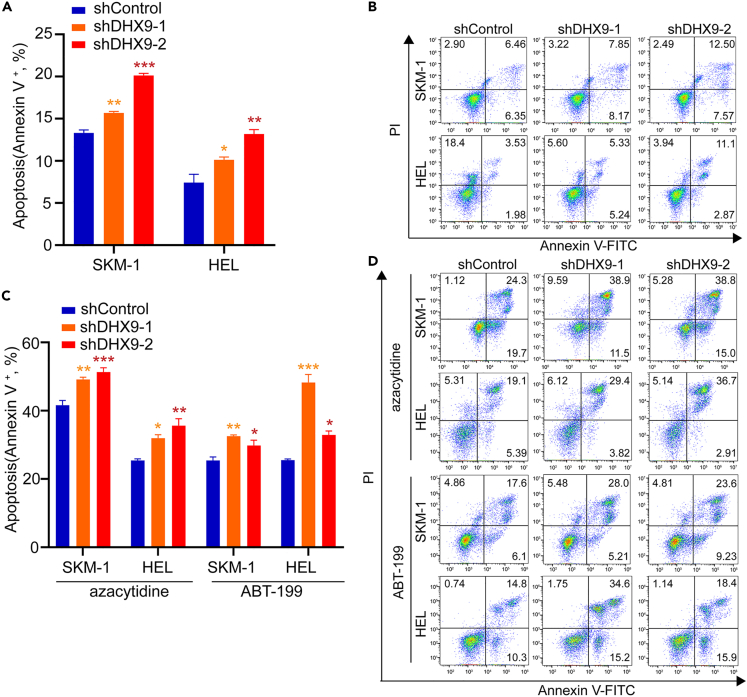
*DHX9* suppression increases cell apoptosis and causes hypersensitivity to chemotherapeutic agents (A and B) Knockdown of *DHX9* increased cell apoptosis in SKM-1 and HEL cells. (C and D) Apoptosis (Annexin V+) was determined in transfected leukemia cell lines with exposure for 48h to azacytidine (4 μM) and ABT-199 (500 nM). ∗: p < 0.05; ∗∗: p < 0.01; ∗∗∗: p < 0.001 (one-way ANOVA test). Data are represented as mean ± SEM.

It is well-known that MDS cells could overcome apoptosis, obtain a proliferative advantage, and then evolve into AML cells. To investigate whether MDS cells overcome apoptosis through DHX9, annexin V-FITC/PI assay was performed and then we observed that knockdown of *DHX9* increased the apoptosis in leukemia cell lines (Figure 3A). The representative flow graphics are shown in Figure 3B. Further, to understand the role of DHX9 in apoptosis triggered by chemotherapeutic agents azacytidine and ABT-199, we investigated the apoptosis sensitivity of leukemia cells to these agents based on *DHX9* knockdown. As shown in Figures 3C and 3D, we observed that the SKM-1 and HEL cells with *DHX9* knockdown showed a significant increase in cell apoptosis after treated with azacytidine and ABT-199. Taken together, the suppression of *DHX9* increases cell apoptosis and caused leukemia cells to be more sensitive to apoptosis induced by chemotherapeutic agents.

### Identification of DHX9-regulated pathways based on RNA sequencing

**Figure 4:**
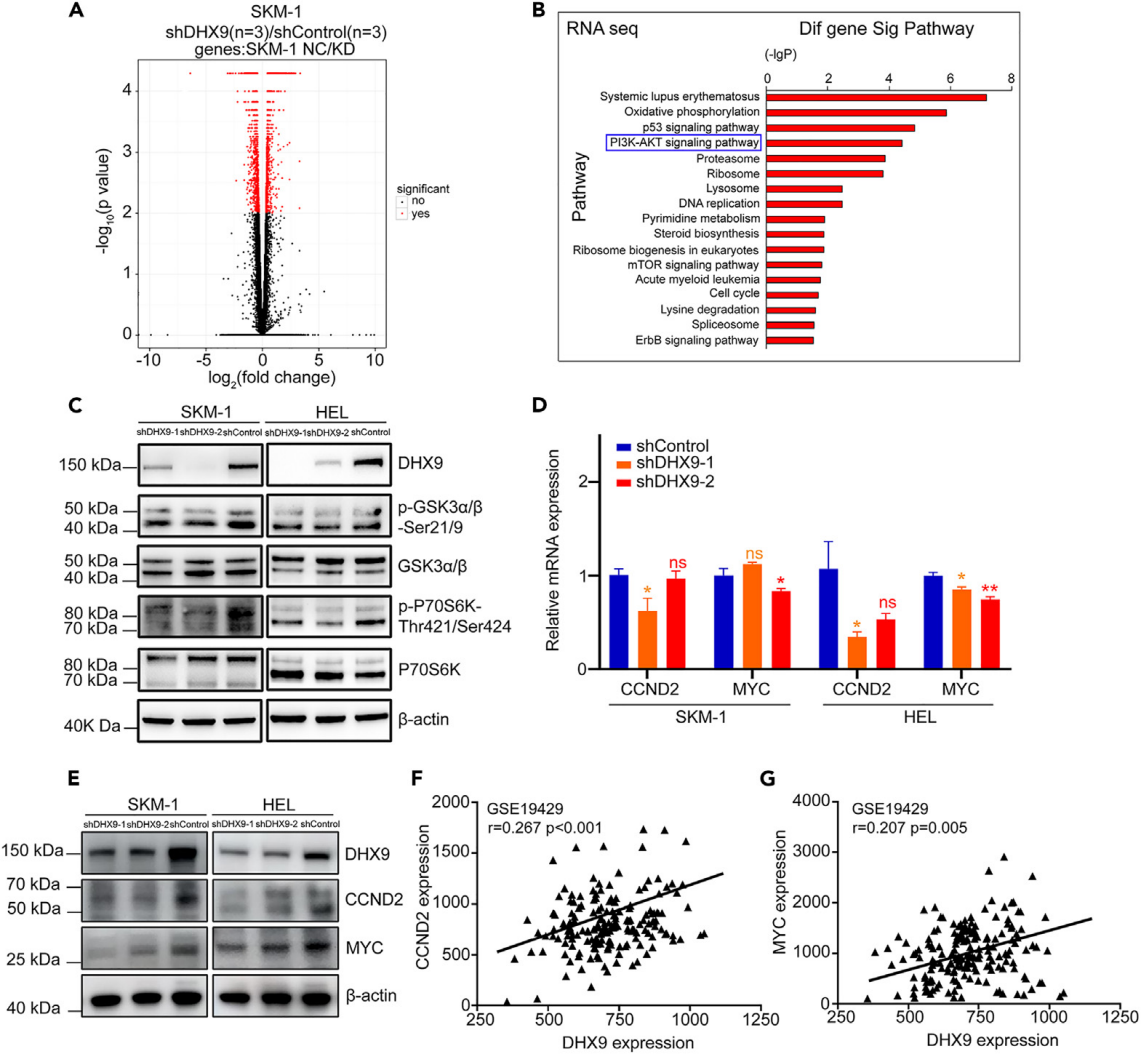
Identification of DHX9-regulated pathways based on combined analysis (A) 3,793 differential genes are identified in *DHX9*-depleted SKM-1 cells compared to the control cells through RNA sequencing. See also Data S1. (B) Pathway analysis of differential genes in SKM-1 cells with *DHX9* knockdown. Integrated analysis suggests that DHX9 may affect the PI3K-AKT signaling pathways. (C) Suppression of *DHX9* decreased the phosphorylation level of GSK3α/β-Ser21/9 and P70S6K-Thr421/Ser424 in leukemia cell lines. (D and E) qRT-PCR and western blotting showing that *DHX9* knockdown leads to reduced expression of CCND2 and MYC in SKM-1 and HEL cells. (F and G) GEM analysis from GEO database (GSE19429) indicated that the expression of *DHX9* had positive correlation with the expression of *CCND2* (F) and *MYC* (G) in the patients with MDS. ∗: p < 0.05; ∗∗: p < 0.01; ∗∗∗: p < 0.001 (One-way ANOVA test or Pearson Correlation Analysis). Data are represented as mean ± SEM.

To further explore the mechanism of DHX9 in regulating malignant biological behaviors, we tried to identify the target genes of DHX9 and the related biological pathways through the gene expression profiles (GEP) from RNA sequencing. We performed RNA sequencing in *DHX9*-depleted SKM-1 cells and control cells and identified 3,793 differential genes (Figure 4A and Data S1). Then, we performed pathway analysis of these differential genes. Significant pathways in SKM-1 included oxidative phosphorylation, P53 signaling, PI3K-AKT pathway signaling, etc. (all p < 0.05) (Figure 4B).

### *Knockdown of DHX9* inhibits the activation of the PI3K-AKT signaling pathway

As described above, the association between DHX9 and its potential pathways such as PI3K-AKT needs to be validated. Compared with the control cells, the *DHX9*-depleted SKM-1 and HEL cells showed significantly decreased phosphorylation level of GSK3α/β-Ser21/9 and P70S6K-Thr421/Ser424 (Figure 4C). *DHX9* knockdown also led to reduced expression of PI3K-AKT signaling-targeted genes such as CCND2 and MYC (Figures 4D and 4E). To validate these data in clinical samples, we analyzed the expression information of *CCND2* and *MYC* in 183 MDS patients (GSE19429) from The Gene Expression Omnibus (GEO) database. Analysis of correlation indicated that *DHX9* expression had a positive relationship with *CCND2* (Figure 4F) and *MYC* (Figure 4G) expression in MDS patients. Our data and GEO data suggested that suppression of *DHX9* significantly inhibits the PI3K-AKT signaling pathway in cell lines and MDS patients. Taken together, DHX9 is indispensable for the activation of PI3K-AKT pathway.

### Defects in *DHX9* promotes R-loop-dependent DNA damage

**Figure 5:**
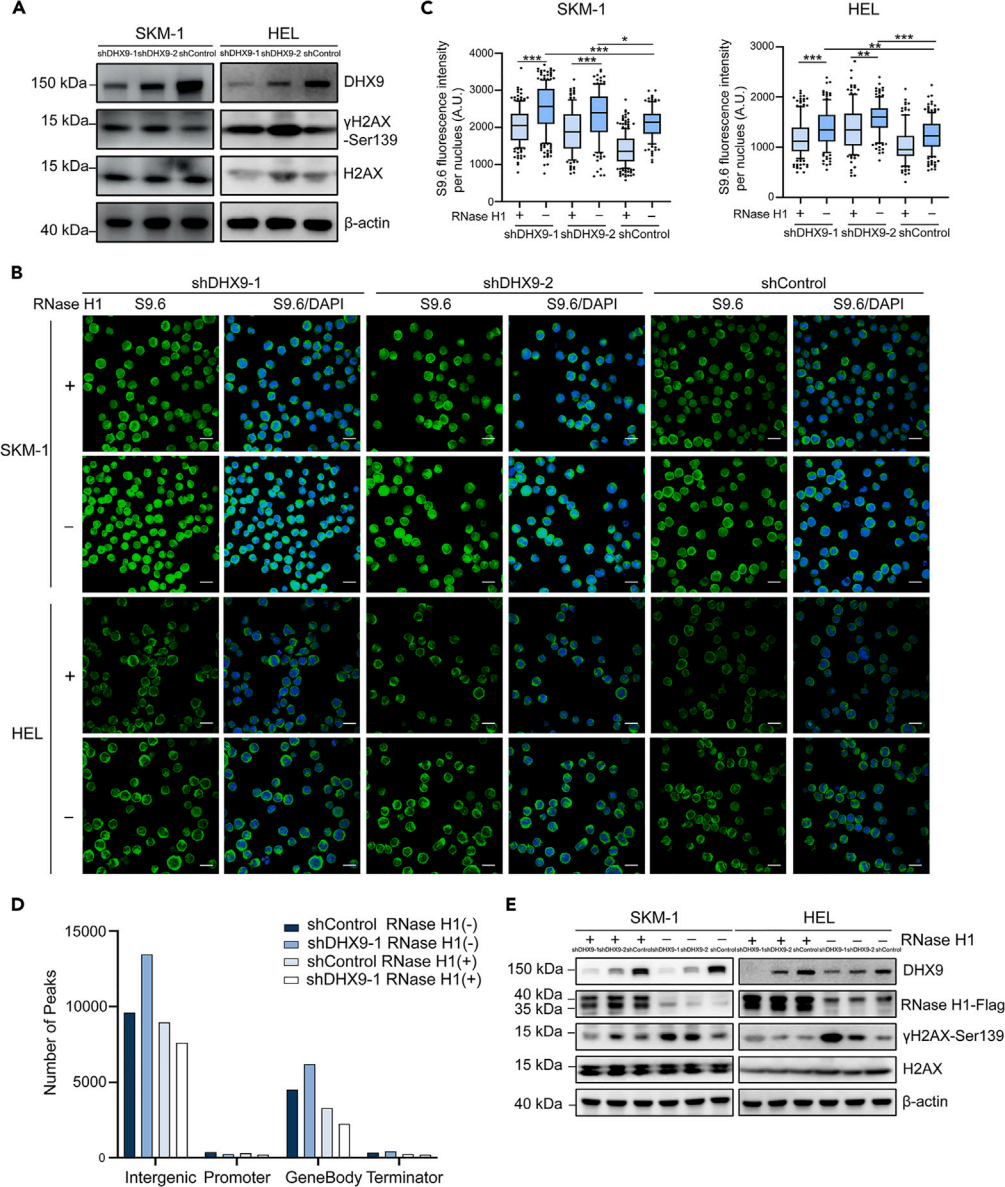
Defects in *DHX9* promotes R-loop-dependent DNA damage (A) Western blotting showing that activation of H2AX phosphorylation induced upon *DHX9* knockdown. (B) Immunostaining with S9.6 antibody (green) and DAPI (blue) in cells transfected with the indicated lentivirus. (C) Intensities of S9.6 staining in individual cells were measured (≥110 nuclei were analyzed by ImageJ). The box represents the median and the 25–75 percentile, whiskers were set to 10–90 percentile, dots represent outliers. Scale bars, 20 μm, AU, arbitrary units. Statistical significance was determined using one-way ANOVA. (D) R-loop peaks enrichment in various gene features by performing DRIP-seq (Intergenic/Promoter [-2/+1 kb around TSS]/GeneBody/Terminator [-1/+2 kb around TES]). (E) The phosphorylation of H2AX induced upon *DHX9* knockdown can be partially rescued by overexpression of RNase H1. ∗: p < 0.05; ∗∗: p < 0.01; ∗∗∗: p < 0.001 (One-way ANOVA test). Data are represented as mean ± SEM.-.

The PI3K-AKT signaling pathway plays an important role in maintaining genomic stability by involving in DNA replication and cell cycle regulation.^13^ It is reported that PI3K inhibition induces DNA damage through nucleoside depletion.^14^ Moreover, impaired PI3K-AKT signaling pathway can inhibit DNA double-strand break (DSB) repair, increase radiation-induced DSB, and improve radiosensitivity.^15^ Based on the above findings, we evaluated the DNA damage by detecting the level of phosphorylated histone H2AX (γH2AX). The results showed that knockdown of *DHX9* increased γH2AX expression in SKM-1 and HEL cells (Figure 5A), which led us to further investigate the role of *DHX9* in this process.

Recently, R-loop accumulation has been proved to be potential mechanism of MDS, which comprises an RNA/DNA hybrid and displaced single-stranded DNA and plays a vital role in the maintenance of genomic stability and cancer development.^16^^,^^17^^,^^18^ Quantitative mass spectrometry (MS)-based proteomics has been used to detect proteins associated with R-loop.^19^ Helicases including DHX9, DDX41, DDX5 were candidate proteins of the R-loop interactome.^20^^,^^21^^,^^22^ Thus, we then explored whether the DNA damage was associated with R-loop formation in *DHX9*-knockdown cells. We performed immunofluorescence with the S9.6 antibody to identify and quantify the RNA-DNA hybrid component of R-loop. *DHX9* suppression increased the level of RNA-DNA hybrids that were sensitive to RNase H1 overexpression (Figures 5B and 5C). Further, we carried out DNA/RNA immunoprecipitation coupled to sequencing (DRIP-seq) and observed that R-loop was enriched in most type of genic regions including intergenic, gene bodies, and terminal regions in *DHX9*-depleted SKM-1 cells (Figure 5D). The signal of R-loop peaks was sensitive to RNase H1 overexpression (Figure 5D). Western blotting showed that RNase H1 overexpression can rescued the impact of *DHX9* suppression on γH2AX in some degree (Figure 5E), revealing that defects in *DHX9* promotes R-loop-dependent DNA damage.

### DHX9 was required for ATR-Chk1 activation

**Figure 6:**
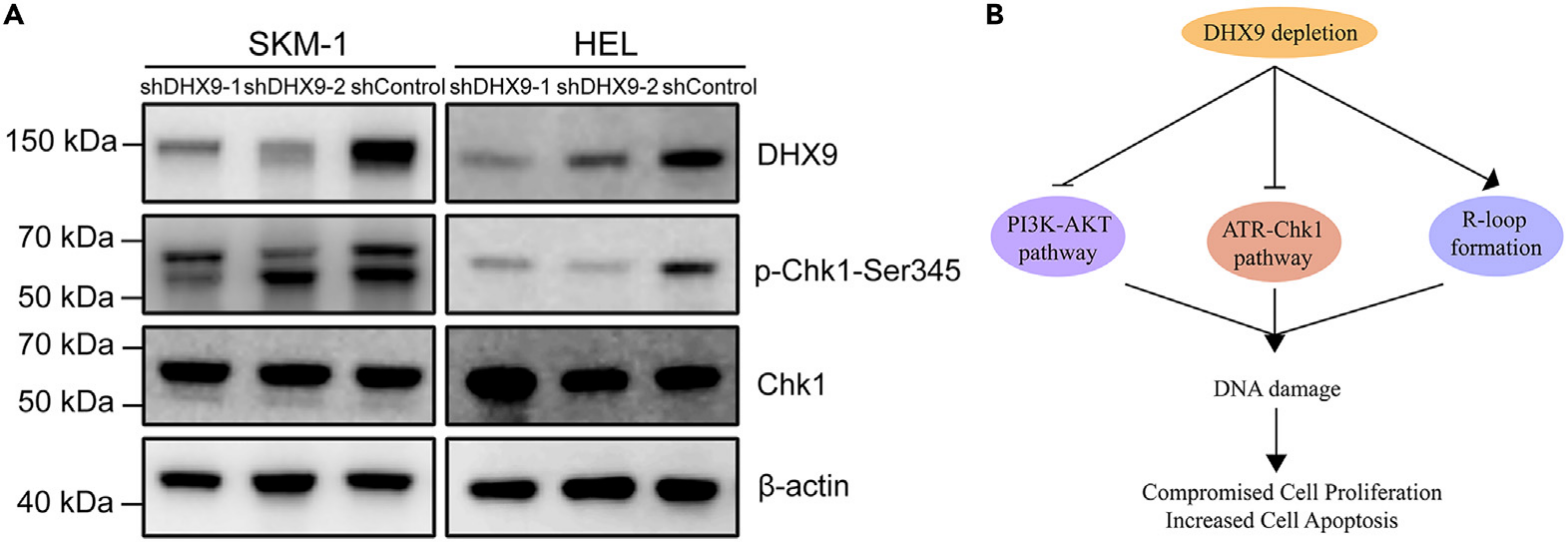
DHX9 was required for ATR-Chk1 activation (A) Western blotting showing that knockdown of DHX9 inhibits phosphorylation of Chk1 on ser 345. β-actin was used as a reference protein. (B) *DHX9* depletion inhibits PI3K-AKT and ATR-Chk1 pathway, induced R-loop formation, finally increased DNA damage, cell apoptosis and compromised cell proliferation.

Previous research have reported that pathological R-loop formation elicited ATR response in MDS with splicing factor mutation.^17^ To determine whether R-loop induced by *DHX9* knockdown can activate ATR-Chk1 pathway, we measured the level of phosphorylated Chk1 (p-Chk1) in cells and found that p-Chk1 expression level diminished in *DHX9*-knockdown cells (Figure 6A). The results showed that DHX9 was required for activation of ATR-Chk1 pathway. In addition, we implied that *DHX9* suppression may further increase DNA damage through inhibiting ATR-Chk1 pathway.

## Discussion

DHX9 is a member of the DEAH helicase family and plays important roles in various biological processes such as DNA replication and RNA processing.^23^^,^^24^ DHX9 interacts with cancer-associated genes such as *CBP*, *BRCA1* and *EGFR* to regulate their transcription and translation.^25^^,^^26^^,^^27^ Studies have shown that the expression level of *DHX9* mRNA was significantly increased in a variety of cancers, such as ewing sarcoma, colorectal cancer, and hepatocellular carcinoma.^28^^,^^29^^,^^30^ Recent studies have reported the role of several *DHX9*-like genes including *DDX41*, *DDX3X,* and *DHX32* in hematological diseases.^31^^,^^32^^,^^33^ These reports suggest that the RNA helicase family may play a vital role in the development of hematological tumors.

In this study, we investigated the clinical significance and biological function of DHX9 in MDS for the first time. We determined the expression of *DHX9* in MDS patients and performed a series of functional experiments to investigate the effects of DHX9 on biological characteristics of MDS/leukemia cells. Higher *DHX9* expression was observed in MDS patients compared with the control individuals and associated with poor survival and high AML transformation, suggesting the potential role of *DHX9* as an oncogene in promoting clonal proliferation in MDS. *In vitro* experiments, DHX9 was found to be essential for the proliferation of leukemia cells, and the knockdown of *DHX9* reduced cell growth, induced cell apoptosis and sensitivity to chemotherapeutic drugs. These results may explain why the MDS patients with *DHX9* overexpression show high AML transformation and suggest that *DHX9* acts as an oncogene in MDS disease progression. The oncogenic characteristic of *DHX9* was also reported in other studies.^34^^,^^35^^,^^36^ A recent study showed that inhibition of *DHX9* expression is lethal to human cancer cell lines and lymphoma cells, while its suppression rarely affected the normal tissues.^37^ *DHX9* family members, such as *DDX1*, *DDX3,* and *DDX5* are considered as oncogenes to promote proliferation of tumor cells.^38^^,^^39^^,^^40^

Mechanistically, DHX9 is indispensable for the activation of PI3K-AKT signaling in this study. Similar to our findings, DHX9 family member DDX5 was also a crucial regulator for activation of AKT signaling in oncogenesis.^40^^,^^41^ Previous studies exhibited the PI3K-AKT signaling pathway was over-activated and associated with disease progression in high-grade MDS.^42^^,^^43^ DHX9 maintains the activation of PI3K-AKT signaling, which explains the reasons that overexpression of *DHX9* is associated with poor survival and high risk of AML transformation in MDS patients.

Genomic instability is one of the hallmarks of tumors; various endogenous and exogenous stress can induce DNA damage and lead to genomic instability. In order to pass on correct genetic information and survive, cells have developed extremely harmonious network called the DNA damage response (DDR).^44^ Previous studies have revealed that DHX9 is a DDR protein, both DHX9 and PI3K have effects on DNA damage repair pathways.^15^^,^^45^^,^^46^ These results enabled us to further explore the function of DHX9. Besides, helicases involving DHX9 have been proved to be interacting with R-loop.^20^^,^^21^ R-loop is three-stranded structure that generates during transcription constantly and is involved in the development of many cancers.^47^^,^^48^^,^^49^ Dr. Nguyen et al. and Dr. Chen et al. revealed separately that splicing factor mutations such as U2AF1 and SRSF2 lead to accumulation of R-loop in MDS.^16^^,^^17^ Accumulation of R-loops impairs transcription, causes replication stress and chromosome fragility, which are linked to compromised proliferation of MDS progenitor cells. A recent survey revealed the ability of DDX47 to unwind R-loop, while another study showed that restoring DDX5 levels in hypoxia lead to further increased replication stress and R-loop.^22^^,^^50^ Our study indicated that knockdown of *DHX9* promotes R-loop accumulation and R-loop-mediated replication stress. Given that R-loop accumulates frequently upon transcription-replication collisions, and *DHX9*-depleted cells had reduced DNA synthesis, we speculated that DHX9 may alleviate transcription-replication conflicts to maintain genomic stability.

ATR is PI3K-related protein kinases and central regulators of the DDR.^51^ Once activated, ATR phosphorylates a variety of substrates particularly Chk1, so as to arrest the cell cycle, stabilize and repair stalled replication forks.^52^ Studies reported pathological R-loop formation elicited ATR response in MDS with splicing factor mutation.^17^ However, Dr. Chakraborty et al. indicated that knockdown of *DHX9* decreased the level of p-ATR and p-Chk1 in U2OS cells.^46^ Consistent with their findings, we investigated p-Chk1 expression was diminished in *DHX9*-depleted MDS/leukemia cells, suggesting that DHX9 maintain ATR-Chk1 activity in response to DNA damage.

Finally, in high-grade MDS, overexpression of *DHX9*, on the one hand, may promote excessive proliferation, and on the other hand, may produce apoptotic resistance to exogenous anti-tumor agents. It is well-known that high-grade MDS and MDS-derived AML show multi-drug resistance. It could be imagined that the application of chemotherapeutic agents in combination with a DHX9 inhibitor will greatly improve the therapeutic effect in MDS.

### Conclusions

In summary, overexpression of *DHX9* is frequent and associated with poor survival and more risk of AML transformation in MDS. Mechanically, DHX9 maintains the activation of PI3K-AKT and ATR-Chk1 pathways, suppress R-loop accumulation and R-loop-mediated DNA damage, and eventually induced excess cell proliferation and apoptosis resistance (Figure 6B). DHX9 may serve as a novel prognostic marker for AML transformation and therapeutic target in MDS.

### Limitations of the study

Although we revealed the connection among *DHX9*, R-loop, PI3K-AKT, and ATR-Chk1 pathway in MDS/leukemia cell lines, the reciprocal relationship among them needs to be further investigated.

## STAR★Methods

### Key resources table

**Table:** 

REAGENT or RESOURCE	SOURCE	IDENTIFIER
**Antibodies**		

DHX9	Abcam	Cat#ab183731, RRID: AB_2868586
GSK3α/β	Cell Signaling	Cat# 5676, RRID: AB_10547140
phospho-GSK3α/β (Ser21/9)	Cell Signaling	Cat# 8566, RRID: AB_10860069
p70S6K	Cell Signaling	Cat# 2708, RRID: AB_390722
Phospho-p70S6K (Thr421/Ser424)	Cell Signaling	Cat# 9204, RRID: AB_2265913
γH2AX (Ser139)	Cell Signaling	Cat# 9718, RRID: AB_2118009
H2AX	Cell Signaling	Cat# 7631, RRID: AB_10860771
phospho-Chk1 (Ser345)	Cell Signaling	Cat# 2348, RRID: AB_331212
Chk1	Cell Signaling	Cat# 2360, RRID: AB_2080320
CCND2	Cell Signaling	Cat# 3741, RRID: AB_2070685
MYC	Cell Signaling	Cat# 5605, RRID: AB_1903938
R-loop (S9.6)	Absolute antibody	Cat# Ab01137, RRID: AB_2936195

**Biological samples**		

Bone marrow mononuclear cells	Shanghai Sixth People’s Hospital Affiliated to Shanghai Jiao Tong University School of Medicine	NA

**Chemicals, peptides, and recombinant proteins**		

RPMI Medium 1640	Gibco	Cat# C11875500
Fetal bovine serum	Yeasen	Cat# 40130ES76
RIPA Buffer	Thermo Fisher	Cat# 89900
MethoCult H4435	Stem Cell	Cat# 04435
Azacitidine	Selleck	Cat# S1782
ABT-199	Selleck	Cat# S8048
DAPI	Sigma Aldrich	Cat# D9542
PVDF membrane	Millipore	ISEQ00010

**Critical commercial assays**		

BCA Protein Assay Kit	Thermo Fisher	Cat# 23227
EdU Cell Proliferation Kit	Beyotime	Cat# C0081S
Annexin V-FITC Apoptosis Detection Kit	Beyotime	Cat# C1062S
RNA Purification Kit	EZbioscience	Cat# B004DP
Reverse Transcription Kit	EZbioscience	Cat# A0010GQ
SYBR Green Master Mix	EZbioscience	Cat# A0012-R2

**Deposited data**		

Expression data from MDS	Pellagatti et al.^53^ and Gorombei et al.^54^	GEO: GSE19429
RNA sequencing data	See Data S1	NA

**Experimental models: Cell lines**		

HEL	ATCC	Cat#TIB-180
SKM-1	Nakagawa et al.^55^	NA

**Oligonucleotides**		

Primer sequences	See Table S2	NA
shRNA sequences	See Table S3	NA

**Software and algorithms**		

IBM SPSS Statistics 25	IBM SPSS Statistics Software	https://www.ibm.com/cn-zh/products/spss-statistics
GraphPad prism 8	GraphPad prism	https://www.graphpad.com/scientific-software/prism/
ImageJ	NIH ImageJ	https://imagej.nih.gov/ij/
Illustrator CC	Adobe	https://www.adobe.com/

### Resource availability

#### Lead contact

Further information and requests for resources and reagents should be directed to and will be fulfilled by the lead contact, Feng Xu (xvfeng3619@163.com)

#### Materials availability

The nucleotide sequences of the shRNA and primer used are available from the lead contact without restriction.

### Experimental model and subject details

#### Patients and cells

120 MDS patients (58 females, 62 male) between 11-84 years of age and 42 non-MDS controls (22 female, 20 male) between 24-91 years were included in this study. No significant difference was observed in age or gender between MDS and control groups. All the participants were East Asians. MDS was diagnosed in accordance with the minimum diagnostic criteria.^56^ The classification and prognostic risk scoring of MDS were performed according to the WHO criteria and the Revised International Prognostic Scoring System (IPSS-R).^57^^,^^58^ Bone marrow mononuclear cells (BMMCs) at the diagnosis time of MDS were obtained for *DHX9* gene expression. All the subjects provided written informed consent for genetic analysis under a protocol approved by the Ethics Committee of Shanghai Sixth People’s Hospital Affiliated to Shanghai Jiao Tong University School of Medicine. HEL cell lines were purchased from ATCC. The SKM-1 cells were a gift from Prof. Nakagawa.^55^ The cells were authenticated using short tandem repeat profiling and tested regularly for Mycoplasma infection.

### Method details

#### RNA preparation and quantitative PCR

The total RNA was extracted using the RNA Purification Kit (EZBioscience, CN) and was reverse transcribed into cDNA. The PCR reactions were performed using the QuantStudio^TM^ 7 Flex System (Thermo Fisher Scientific, US) and SYBR Green Master Mix (EZBioscience, CN). The relative expression of genes was calculated using 2^−ΔΔCT^. GAPDH was used as a reference gene. All primers information was shown in Table S2.

#### Lentivirus-mediated cell transfection

Two *DHX9*-shRNAs and non-specific shControl sequences were cloned into the hU6-MCS-CMV-Puromycin vector, while RNase H1 CDS sequence was cloned into the Ubi-MCS-3FLAG-SV40-Neomycin vector (JIKAI, Shanghai). The lentivirus package was performed in HEK 293T cells. In brief, 5×10^5^ cells/well in a 6-well plate was incubated with the lentivirus (3×10^8^ TU/mL) and polybrene (5 μg/ml) in a 1 mL volume. The silencing efficiency of the *DHX9* was evaluated using qPCR and western blotting. The sequences of shRNA were shown in Table S3.

#### Cell Counting Kit-8 assay

Cell proliferation was evaluated by Cell Counting Kit-8 (CCK-8) assay at different time point after plating in 96-well plates (4,000 cells/well) in triplicate. The cell growth curve was described according to the absorbance values at 450nm.

#### EdU proliferation assay

5-ethynyl-2′-deoxyuridine (EdU) uptake assay was also used to determine the cell proliferation. In briefly, cells transfected with sh*DHX9* or shControl were incubated with EdU (10 μM) for 2 hours in complete medium. After treatment with a fixing solution and a permeabilizing solution, the cells were stained with anti-EdU antibody and Propidium Iodide (PI). EdU staining was performed with BeyoClick™ EdU Cell Proliferation Kit with Alexa Fluor 647 (Beyotime, CN), according to the manufacturer’s protocol. Stained cells were run on a FACS Calibur (Beckman, US). The data were analyzed using FlowJo 10.6.2 software.

#### Colony formation assay

Cells were plated in 35mm dishes with methylcellulose medium (MethoCult™ H4435) containing SCF, GM-CSF, IL-3, and erythropoietin (Stem Cell Technologies, CA) at 800 cells/well in triplicate wells for each condition. After 14 days of incubation in a humidified incubator at 37°C, the colonies containing at least 30 cells were counted.

#### Apoptosis detection

Annexin V-FITC/PI assay (beyotime, CN) was used to quantitatively detect the cell apoptosis according to the manufacturer’s instructions. Annexin V-FITC positive cells were considered to be apoptotic cells.

#### Measurement of apoptosis-sensitive to apoptosis-inducing agents

SKM-1 and HEL cells transfected with shControl or sh*DHX9* were cultured for 48 hours in the presence and absence of 5-Azacytidine and ABT-199. 5-Azacytidine and ABT-199 were purchased from Selleck Chemicals (US). Cell apoptosis was analyzed using Annexin V-FITC/PI.

#### RNA sequencing

cDNA libraries were prepared using TruSeq RNA Sample Preparation Kit (Illumina), according to the manufacturer’s protocol. One microgram of total RNA was used for sequencing on a HiSeq 2000 instrument (Illumina) with 100 bp paired end reads, according to the manufacturer’s protocol. Data analysis was performed using edgeR27 to evaluate the whole transcript expression (false discovery rate <0.05) and with DEXSeq28 to evaluate differential exon usage (false discovery rate <0.05). The GEPs were identified between the SKM-1 cells with *DHX9* knockdown (n = 3) and the control cells (n = 3).

#### Western blotting

The following antibodies were used for western blotting: DHX9 (Abcam, ab183731), GSK3α/β (Cell Signaling, #5676), p-GSK3α/β-Ser21/9 (Cell Signaling , #8566), P70S6K (Cell Signaling, #2708), p-P70S6K-Thr421/Ser424 (Cell Signaling, #9204), γH2AX(Cell Signaling, #9718), H2AX(Cell Signaling, #7631), p-Chk1 (Cell Signaling, #2348), Chk1 (Cell Signaling, #2360), Cyclin D2 (Cell Signaling, #3741), Myc (Cell Signaling, #5605), β-actin (Share-bio, SB-AB2001), secondary anti-rabbit IgG antibody (Proteintech, SA00001-2), secondary anti-mouse IgG antibody (Proteintech, SA00001-1). The detailed protocol is described in a previous study.^8^

#### Immunofluorescence

The cells were fixed in 4% paraformaldehyde (PFA) for 20 min, then washed once with PBS and transfer to glass slides. Subsequently, cells were permeabilized with 0.5% Triton X-100 and blocking buffer for 30 min successively, and then incubated with S9.6 antibody (Absolute antibody, Ab01137) overnight at 4 °C. After incubation, cells were washed three times in PBS and incubated with Alexa Fluor-488 conjugated secondary antibody (Cell Signaling Technology, #4412) for 1 hour. The nuclei were stained with 1 ug/ml DAPI (Sigma-Aldrich, D9542) for 10 min. Images were acquired by using laser scanning confocal microscopy.

#### DRIP-seq analysis

The total nucleic acids were extracted from the SKM-1 cells transfected with lentivirus. DRIP was immunoprecipitated with S9.6 antibody and IP efficiency was assessed by qPCR. The qualified libraries were sequenced on an Illumina NovaSeq 6000 system. The 5′, 3′-adaptor bases were trimmed using cutadapt software. The trimmed reads were aligned to human reference genome GRCh38 using bowtie2 software. The aligned reads were used for peak calling of the DRIP regions using MACS2. Statistically significant DRIP-enriched peaks were identified at a p-value threshold of 0.001. The peaks were annotated with the overlapping or nearest gene. Differentially accessible peaks were analyzed using DiffBind.

### Quantification and statistical analysis

Statistical analysis were conducted using SPSS software version 25.0 and Graphpad Prism 8. The association of mutations with clinical characteristics was analyzed by the chi-squared (χ2) test. The Kaplan-Meier test was used for univariate survival analysis. Two independent samples were compared using Student’s *t*-test. Three independent samples were compared using one-way ANOVA. The results were considered statistically significant with a p-value less than 0.05. The corresponding statistical analysis relative to the control group is annotated with an asterisk. ∗: p < 0.05; ∗∗: p < 0.01; ∗∗∗: p < 0.001.

## Data Availability

• The raw RNA-seq data in paper are presented in the Supplementary Materials. The data of gene expression from MDS are available at GEO database with accession number GSE19429. • This paper does not report original code. • Any additional information required to reanalyze the data reported in this paper is available from the lead contact upon request.

## Supplemental information

Table S1. Clinical information of MDS patients, related to Table 1 and STAR Methods

Document S1. Tables S2 and S3

Data S1. Identification of differential genes in SKM-1 cells with *DHX9* knockdown, related to Figure 4 and STAR Methods
