# Supplementary material for: DHX9-mediated pathway contributes to the malignant phenotype of myelodysplastic syndromes
Source: iScience. 2023 May 25;26(6):106962. doi: 10.1016/j.isci.2023.106962 (PMC10250162; doi:10.1016/j.isci.2023.106962)
Supplement: Document S1. Tables S2 and S3 [file mmc2.pdf]

Table S2. Primer sequences for qRT-PCR, related to STAR Methods.

Gene	F/R	Transcript	Sequence
DHX9	F	NM_001357	5'-CAGGAGAGAGAGTTACTGCCT-3'
	R		5'-CTCTGCTGCTCGGTCATTCTG-3'
GAPDH	F	NM_001256799	5'-GGAGCGAGATCCCTCCAAAAT-3'
	R		5'-GGCTGTTGTCATACTTCTCATGG-3'
CCND2	F	NM_001759	5'-ACCTTCCGCAGTGCTCCTA-3'
	R		5'-CCCAGCCAAGAAACGGTCC-3'
MYC	F	NM_002467	5'-GTCAAGAGGCGAACACACAAC-3'
	R		5'-TTGGACGGACAGGATGTATGC-3'

Table S3. shRNA sequences, related to STAR Methods.

NO.	Target Seq
shDHX9-1	gaAGGATTACTACTCAAGAAA
shDHX9-2	agACTTAATATGGCTACACTA aaGCATGGACCTCAAGAATGA
